# A Targeted LC‐MS Strategy for Low‐Abundant HLA Class‐I‐Presented Peptide Detection Identifies Novel Human Papillomavirus T‐Cell Epitopes

**DOI:** 10.1002/pmic.201700390

**Published:** 2018-05-02

**Authors:** Renata Blatnik, Nitya Mohan, Maria Bonsack, Lasse G. Falkenby, Stephanie Hoppe, Kathrin Josef, Alina Steinbach, Sara Becker, Wiebke M. Nadler, Marijana Rucevic, Martin R. Larsen, Mogjiborahman Salek, Angelika B. Riemer

**Affiliations:** ^1^ Immunotherapy and Immunoprevention German Cancer Research Center (DKFZ) Im Neuenheimer Feld 280 69120 Heidelberg Germany; ^2^ Molecular Vaccine Design German Center for Infection Research (DZIF) Partner Site Heidelberg Heidelberg Germany; ^3^ Department of Biochemistry and Molecular Biology University of Southern Denmark Odense M Denmark; ^4^ Division of Stem Cells and Cancer German Cancer Research Center (DKFZ) and Heidelberg Institute for Stem Cell Technology and Experimental Medicine (HI‐STEM) Heidelberg Germany; ^5^ Massachusetts General Hospital Center for Cancer Research Charlestown MA USA

**Keywords:** human papillomavirus (HPV), immunopeptidomics, immunotherapy, neoepitopes, targeted mass spectrometry (MS)

## Abstract

For rational design of therapeutic vaccines, detailed knowledge about target epitopes that are endogenously processed and truly presented on infected or transformed cells is essential. Many potential target epitopes (viral or mutation‐derived), are presented at low abundance. Therefore, direct detection of these peptides remains a challenge. This study presents a method for the isolation and LC‐MS^3^‐based targeted detection of low‐abundant human leukocyte antigen (HLA) class‐I‐presented peptides from transformed cells. Human papillomavirus (HPV) was used as a model system, as the HPV oncoproteins E6 and E7 are attractive therapeutic vaccination targets and expressed in all transformed cells, but present at low abundance due to viral immune evasion mechanisms. The presented approach included preselection of target antigen‐derived peptides by in silico predictions and in vitro binding assays. The peptide purification process was tailored to minimize contaminants after immunoprecipitation of HLA‐peptide complexes, while keeping high isolation yields of low‐abundant target peptides. The subsequent targeted LC‐MS^3^ detection allowed for increased sensitivity, which resulted in successful detection of the known HLA‐A2‐restricted epitope E7_11–19_ and ten additional E7‐derived peptides on the surface of HPV16‐transformed cells. T‐cell reactivity was shown for all the 11 detected peptides in ELISpot assays, which shows that detection by our approach has high predictive value for immunogenicity. The presented strategy is suitable for validating even low‐abundant candidate epitopes to be true immunotherapy targets.

## 1 Introduction

Immunotherapies targeting tumor‐specific antigens represent attractive cancer treatments, as they allow focusing the immune attack specifically on cancer cells. Truly tumor‐specific antigens can either derive from oncogenic viruses or from tumor‐specific mutations (so‐called neoantigens). To be visible to the immune system, peptide epitopes derived from these antigens must be presented on the cell surface on human leukocyte antigen (HLA) molecules for T‐cell recognition.^1^

Cytotoxic CD8^+^ T‐cells recognize epitopes presented on HLA class‐I (HLA‐I). HLA‐I is a heterodimeric membrane glycoprotein composed of an α‐chain and the non‐covalently bound β_2_‐microglobulin (β_2_M). The HLA‐I complex typically binds short 8–11‐mer peptides. Epitopes are generated from the whole cell proteome by the cellular antigen processing machinery, resulting in diverse epitope sequences of various abundances presented at the cell surface.^2^ HLA‐I molecules are highly polymorphic, and each type binds different peptides based on the respective anchor motifs.^3^, ^4^ In this study, we investigated HLA‐A2‐restricted peptides since HLA‐A2 is the most common HLA allele worldwide.^5^

Due to the expression of viral proteins, human papillomavirus (HPV)‐mediated cancers are among the tumors that can be targeted by immunotherapies. High‐risk types of HPV cause anogenital and oropharyngeal cancer. The most important HPV‐induced malignancy is cervical cancer, which is the fourth most common cancer in women worldwide.^6^ The vast majority of cervical cancer is caused by HPV16 and HPV18 (60 and 15% of cases, respectively).^6^, ^7^ The likelihood to get infected with a high‐risk HPV type and consequently develop cancer was reduced after prophylactic vaccines became available for immunization of adolescents in 2006.^8^ However, these vaccines have no therapeutic effects in already infected individuals. It has been shown that T‐cell responses against HPV‐derived epitopes effectively cleared HPV infections.^9^ Thus, immunotherapeutic strategies targeting the HPV oncoproteins E6 and E7, which are expressed in all stages of HPV‐mediated carcinogenesis, are actively pursued to induce effective clearance of HPV in infected individuals.^10^, ^11^ One possible approach is direct identification of HPV‐derived epitopes that are naturally presented on the surface of HPV‐transformed cells, and developing therapeutic vaccinations based on these validated targets. To date, several HLA‐A2‐restricted HPV16 target epitopes were identified with indirect biological assays.^12^ However, only one HLA‐A2‐restricted HPV16 peptide, E7_11–19_, was directly detected on the surface of HPV16^+^ cell lines^13^ and primary tumors.^14^ This is probably due to the fact that the amounts of HPV epitopes presented on the cell surface are extremely low, because of low viral protein expression and known HPV immune evasion mechanisms.^15^, ^16^

**Significance Statement**

This study describes the direct LC‐MS^3^ detection of 11 out of 17 preselected HLA‐A2‐restricted HPV16 E6 and E7 peptides on the surface of HPV‐transformed cells, and shows immunogenicity of all of these. Interestingly, half of the detected epitopes only have moderate or weak binding affinities to HLA‐A2 molecules. This shows that not only strong binding HPV16‐derived peptides but also weak HLA binders are presented to T‐cells for immune recognition. Our results contribute to a better understanding of epitope presentation in HPV‐induced cancers and the detected peptides will be used for future immunotherapy development. Furthermore, the described strategy can be applied for the detection of other low‐abundant epitopes, such as mutation‐derived neoepitopes, which are usually missed with untargeted LC‐MS^2^ acquisition.

Also epitopes derived from other viral proteins and many neoepitopes are presented at low abundance, and thus pose challenges to direct identification approaches. Therefore, the aim of the present study was to develop a sample preparation strategy that allowed high isolation yields of low‐abundant target peptides combined with a targeted LC‐MS detection strategy with two consecutive MS fragmentation stages (MS^3^), which would allow considerably higher sensitivity necessary for low‐abundant peptide detection.^17^

## 2 Experimental Section

*In Silico Predictions*: In silico predictions for HLA‐A*0201 binding were performed for HPV16 E6 and E7 proteins with 11 web‐accessible algorithms. The predictions were performed for 8–11‐mer peptides with NetMHC 4.0,^18^ NetMHC 3.4,^19^ NetMHCpan 3.0,^20^ NetMHCpan 2.8,^20^ NetMHCcons 1.1,^21^ Consensus,^22^ PickPocket 1.1,^23^ SMM,^24^ SMMPMBEC,^25^ BIMAS,^26^ and SYFPEITHI.^27^ Results of all prediction algorithms were combined. To ensure not to miss potential binders at this stage, after starting with the recommended cut‐offs of the respective algorithm, the prediction cut‐offs were lowered stepwise based on actual binding of tested peptides in the in vitro binding assays, until no more binders could be detected.

*Cell Lines, Media*: Adherent HLA‐A2^+^ HPV16^+^ CaSki cervical cancer cells (ATCC CRL‐1550) and suspension HPV16‐negative HLA‐A2^+^ BSM cells (IHWG Cell Bank) were used in this study. CaSki cells were cultivated in RPMI‐1640 medium supplemented with 10% heat‐inactivated FCS and 1% penicillin/streptomycin (all Sigma‐Aldrich, Taufkirchen, Germany). BSM cells were cultivated in RPMI‐1640 supplemented with 15% heat‐inactivated FCS and 1 mm sodium pyruvate (PAA Laboratories, Cölbe, Germany). Cells were kept under standard conditions in a humidified incubator at 37 °C and 5% CO_2_. They were authenticated^28^ and regularly checked for contaminations by multiplex PCR^29^ by the DKFZ Genomics and Proteomics core facility.

*Synthetic Peptides*: All synthetic peptides used in this study were produced with a purity of >95% at the DKFZ peptide production unit. Peptides were dissolved in DMSO at 10 mg mL^−1^ and stored in small aliquots at −80 °C.

*In Vitro Competition Based Binding Assays*: In vitro competition‐based binding assays were performed as described in ref. 30. In brief, BSM cells were treated with citric acid buffer to strip off naturally presented peptides. Cells were incubated overnight at 4 °C with β2M, a constant concentration (150 nm) of fluorescein‐labeled HLA‐A2‐binding reference peptide (FLPSDC(Fl)FPSV), and six different concentrations (0.78 –100 μm) of the respective investigated non‐labeled peptide, competing for HLA‐binding. Fluorescence was measured by flow cytometry and data processed with FlowJo 10 (TreeStar, Ashland, OR, USA). A logistic four‐parametric non‐linear regression calculation using SigmaPlot 13 (Systat‐Software, San José, CA, USA) was conducted to calculate binding affinity (IC_50_). The IC_50_ value is defined as the test peptide concentration that inhibits binding of the fluorescently labeled reference peptide by 50%. Peptides were classified as strong (IC_50_ < 5 μm), intermediate (IC_50_ 5–15 μm), weak (IC_50_ 15–100 μm), or non‐binders (IC_50_ > 100 μm) according to ref. 30. The assay was performed with at least three biological replicates for binders and two for non‐binders.

*Immunoprecipitation of Peptide/HLA‐A2 Complexes*: 5 × 10^8^ CaSki (for HPV peptide analysis) or BSM cells (for control purposes) were collected by scraping or pelleting, respectively, and lysed with a lysis buffer containing 1.2% CHAPS (AppliChem, Darmstadt, Germany) or 1% IGEPAL CA‐630 (Sigma‐Aldrich), protease inhibitor cocktail (cOmplete‐Mini, Roche, Mannheim, Germany) and 1 mm PMSF (Roth, Karlsruhe, Germany) in PBS. Cell lysates were centrifuged to remove cell debris (22 000 × *g*, 4 °C, 30 min). Peptide/HLA‐A2 complexes were isolated via IP from cleared cell lysates by mouse‐anti‐human‐HLA‐A2 (clone BB7.2) antibody coupled to GammaBind Sepharose beads (GE Healthcare, Uppsala, Sweden) for 4 h at 4 °C. IP samples were washed seven times with 15 mL ice‐cold PBS, transferred to 1.5 mL tubes and washed four times with 1.5 mL ice‐cold PBS.

*Peptide Elution and Purification from IP Samples by Ultrafiltration and Reverse Phase Materials*: Peptides were eluted from isolated peptide/HLA complexes (pHLA) with 0.3% TFA (ProteoChem, Denver, CO, USA) in LC‐MS‐grade water (Biosolve, Valkenswaard, The Netherlands). Three ultrafiltration devices (Vivacon 500 2 kDa or 10 kDa cut‐off, Sartorius Stedim, Göttingen, Germany; Amicon 3 kDa cut‐off, Merck‐Millipore, Cork, Ireland) were used. Peptide RP purification was performed with 1 mL Sep‐Pak tC18 cartridges (Sep‐Pak; Waters, Milford, MA, USA), with in‐house assembled microcolumns in 200 μL pipette tips as in ref. 31, filled with Zorbax SB‐C18 5 μm (Agilent Technologies, Santa Clara, CA, USA) or with OligoR3 50 μm (Applied Biosystems, Bedford, MA, USA) material. The resulting protein fractions were subjected to in‐solution reduction, alkylation, and digestion, and subsequently analyzed by LC‐MS with solid‐phase extraction capillary liquid chromatography (speLC) coupled to a Q‐Exactive instrument. The exact experimental procedures of assessment of peptide purification are given in Materials and Methods, Supporting Information.

*Detergent Removal*: To examine removal of the nonionic detergent IGEPAL CA‐630 from IP samples, ion exchange was performed with microcolumns filled with strong cation exchange or strong anion exchange materials. Target peptide recoveries were assessed by targeted LC‐MS^2^. The efficiency of detergent removal by the material giving the highest target peptide yield was analyzed by MALDI‐TOF MS^1^. Experimental details are provided in Materials and Methods, Supporting Information.

*Detection of HLA‐A2‐Presented Peptides by Targeted LC‐MS^3^ Mass Spectrometry*: The optimized sample preparation steps were applied to IP samples of the HLA‐A2^+^ HPV16^+^ cervical cancer cell line CaSki. All 17 HPV16‐derived peptides that were determined to be HLA‐A2 binders in the cellular binding assays and two endogenous control HLA‐A2 binders (derived from housekeeping proteins) were used for generation of LC‐MS^3^ methods. These 19 peptides were monitored in the CaSki IP samples. A minimum of three biological replicates was measured for each experiment. Reference spectra of synthetic peptides were manually compared by three independent researchers (R.B., N.M., M.S.) to the spectra of IP samples to confirm the presence of target peptides. The criteria for positive identification were as follows: First, retention times for the peptide detected in the IP sample and its corresponding synthetic peptide had to coincide. Second, the extracted ion chromatograms for all transitions (an *m/z* pair of a precursor and a fragment ion) had to be measured concurrently and in correct hierarchy of abundance in IP samples and for the synthetic reference peptides. Finally, MS^3^ spectra were monitored for a minimum of three transitions and were required to match between the synthetic peptide and the peptide identified in the IP sample. Only peptides that were assessed to fulfil all criteria by all three independent researchers were considered to be detected. Detailed MS measuring parameters and data processing specifications are provided in Materials and Methods and Table S1, Supporting Information. Data have been deposited in PeptideAtlas, with the Identifier PASS01152. As PeptideAtlas data are handled by ProteomeCentral, and thus exchanged with PRIDE, our data will also be available to the newly established SysteMHC Atlas project.^32^

*Immunogenicity Assessment by Interferon‐γ ELISpot*: Peripheral blood mononuclear cells (PBMCs) were isolated from buffy coats from anonymous healthy blood donors (obtained from DRK blood bank Mannheim), under the local IRB approval S‐394/2011. We only used buffy coats from female donors >40 years of age, to increase the likelihood of a previous HPV16 encounter. PBMCs were isolated by standard density centrifugation with Ficoll‐Paque Plus (GE Healthcare, Uppsala, Sweden) in Leucosep tubes (Greiner Bio‐One, Frickenhausen, Germany). They were further tested for HLA‐A2 expression by staining with a FITC‐coupled anti‐human HLA‐A2 antibody (clone BB7.2) and subsequent analysis by flow cytometry.

1–2 × 10^6^ HLA‐A2^+^ PBMCs were seeded in RPMI‐1640, supplemented with 10% human serum, 1% penicillin/streptomycin, 2 mm L‐Glutamine, 12 mm HEPES, 0.05 mm 2‐mercaptoethanol (all Sigma‐Aldrich), and 10 ng mL^−1^ IL‐7 (R&D Systems, Minneapolis, MN, USA) in the presence of HPV16‐derived peptides with a final concentration of 10 μg mL^−1^. A HLA‐A2‐restricted HIV1 peptide (LTFGWCFKL‐HIV/Nef^137‐145^) was used as negative control, 1 μg mL^−1^ of the CEF peptide pool (PANATecs, Heilbronn, Germany) as positive control, and 1 μL mL^−1^ DMSO (peptide diluent) as background control. On day 3 and 7, cells were fed with 20 U mL^−1^ IL‐2 (PeproTech, Rocky Hill, NJ, USA). Cells were harvested on day 12, restimulated with the respective peptide or control and seeded in triplicate wells at a density of 1–2.5 × 10^5^ on Multiscreen‐HA ELISpot plates (Merck‐Millipore, Darmstadt, Germany) coated with 2 μg mL^−1^ of anti‐human interferon (IFN)‐γ antibody (clone 1‐D1K). After 24 h, 0.5 mg mL^−1^ biotinylated anti‐human IFN‐γ antibody (clone 7 B6‐1) was used to develop the ELISpot according to the manufacturer's instructions (Mabtech AB, Nacka Strand, Sweden). The number of spots was analyzed with a CTL ELISpot reader. The number of spot‐forming units (SFU) per 1 × 10^6^ cells was determined and expressed relative to the background control (stimulation index, SI). A peptide‐specific T‐cell response was considered positive when SI > 2 and SFU > 200 spots per 1 × 10^6^ cells.

## 3 Results

**Figure 1:**
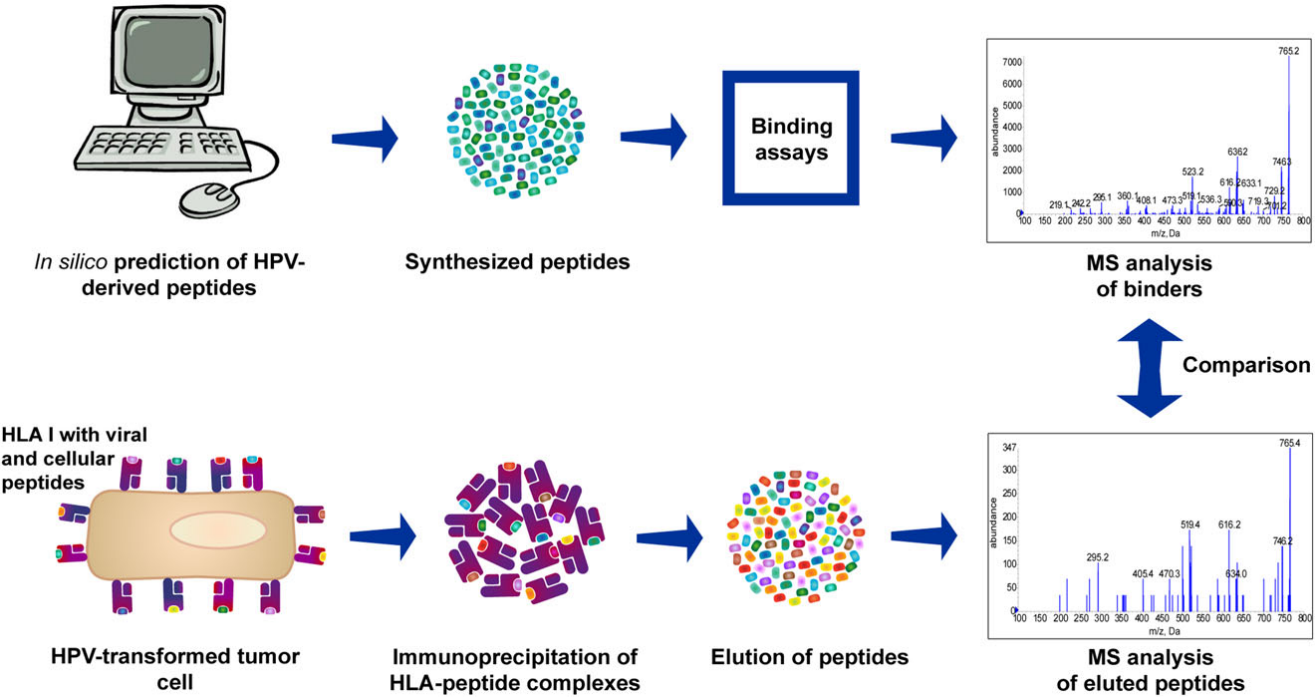
Workflow for direct identification of epitopes presented on the cell surface. HPV16 E6/E7‐derived peptides were predicted in silico for their binding affinities to HLA‐A2. Predicted binders were tested for their actual binding affinity in in vitro assays. Only confirmed binders were monitored by MS analysis after their isolation from HPV‐transformed cells.

The general strategy used in our study is shown in Figure 1. Binding of HPV16‐derived peptides to HLA‐A2 was predicted in silico. Predicted binders were tested for their true binding affinity to HLA‐A2 molecules in competition‐based cellular binding assays. Verified binders were used to design targeted LC‐MS^3^ methods. Subsequently, pHLA were isolated from HPV16^+^ cells via IP, peptides were purified and monitored by LC‐MS^3^ analysis. Detected peptides were assessed for immunogenicity.

### 3.1 HLA‐A2 Ligand Predictions and Cellular Binding Assays

**Figure 2:**
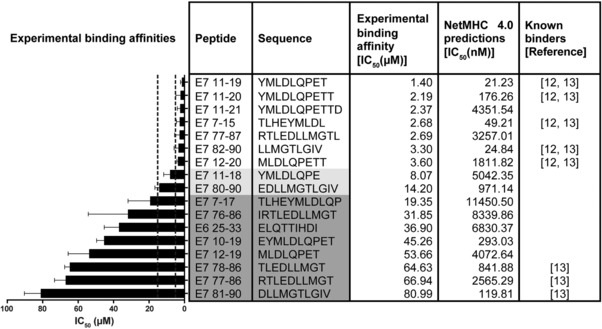
Experimentally determined and predicted binding affinities for HLA‐A2‐restricted HPV16 E6/E7‐derived peptides. In silico predictions were conducted with 11 web‐based algorithms. For clarity, only results from NetMHC 4.0 are displayed. Experimental binding affinities were determined with in vitro competition‐based binding assays. The order of peptides is from strongest (*top*) to weakest (*bottom*) binding affinity as determined in the in vitro assays. Strong binders: experimental IC_50_ below 5 μm; intermediate binders: experimental IC_50_ 5–15 μm (marked with light gray); weak binders: experimental IC_50_ > 15–100 μm (marked with dark gray). Results are plotted as mean ± SD from at least three experimental replicates.

In silico predictions with adapted cut‐offs suggested 121 potential HLA‐A2 binders derived from HPV16 E6 and E7 proteins (data not shown). Cysteine‐containing peptides were excluded, as cysteines are prone to intra‐ and intermolecular disulfide bond formation and therefore may complicate targeted MS analysis. The binding affinity of 58 non‐cysteine‐containing peptides was assessed in competition‐based cellular binding assays. We identified 17 binding peptides, of which 9 were never reported before (Figure 2). Seven peptides were strong, two peptides were intermediate, and eight peptides were weak binders. As also shown in Figure 2, predicted and experimental binding affinities only partially overlap.

### 3.2 Optimization of Sample Preparation

pHLA were isolated from HPV16^+^ CaSki cells by HLA‐A2‐specific IP. As low amounts of target peptides were expected (due to known low expression levels of the viral source proteins), we adapted the IP isolation and epitope extraction to minimize purification steps and peptide losses. In most current epitope isolation workflows, peptides are dissociated from the HLA complex by acidification and further separated from protein contaminants by ultrafiltration. As high peptide losses can be associated with ultrafiltration, we compared several ultrafiltration tubes and RP materials for most efficient peptide extraction. Our data show that the recovery of target HPV16 E6/E7‐derived peptides was better with RP materials than with ultrafiltration (Figure S1, Supporting Information). Next, we tested three RP materials for efficiency of separation of larger proteins from target peptides by differential elution. Sample processing on Zorbax microcolumns resulted in least contaminating protein amounts and best target peptide recoveries (Figure S2, Supporting Information), and was therefore chosen as our standard epitope extraction strategy.

Since the high detergent content in the sample can interfere with LC‐MS analysis, it needs to be removed either directly after IP by extensive washing or during peptide extraction from IP samples. To examine the possibility of removing the nonionic detergent IGEPAL CA‐630 during peptide extraction, we measured peptide recovery from various ion exchange materials. The strong cation exchange material Poros 20HS showed highest peptide yields (Figure S3, Supporting Information), and was thus further tested for detergent removal efficiency. Even though we could demonstrate removal of excess detergent (Figure S4, Supporting Information), the procedure resulted in marked peptide losses. Thus, for detergent removal, extensive washing of the IP sample directly after IP is preferable over additional sample handling steps, and was incorporated in our sample preparation workflow.

### 3.3 Identification of HLA‐A2‐Presented HPV16‐Derived Peptides

We next employed the optimized IP and epitope extraction strategy—ensuring minimal protein and detergent contaminations—to IP samples of HLA‐A2^+^ HPV16^+^ CaSki cells. All peptides that were experimentally identified as HLA‐A2 binders (Figure 2) were used for manual optimization of LC‐MS^3^ methods to assess their presence on HPV16‐positive cancer cells. The identity of target peptides in IP samples was confirmed with several criteria, as detailed in the Experimental section and in the Methods section, Supporting Information. It should be noted that most of the target peptides contained methionine (Met). Detection challenges for these peptides are shown in Figure S5, Supporting Information. Met‐containing peptides were only detected in the oxidized state (MetOx) in the IP samples. Therefore, the MS^3^ spectra of detected MetOx peptides had shifts of 8 or 16 *m/z* (for doubly or singly charged ions, respectively) for all precursor ions and, depending on the sequence, also the majority of fragment ions.

**Figure 3:**
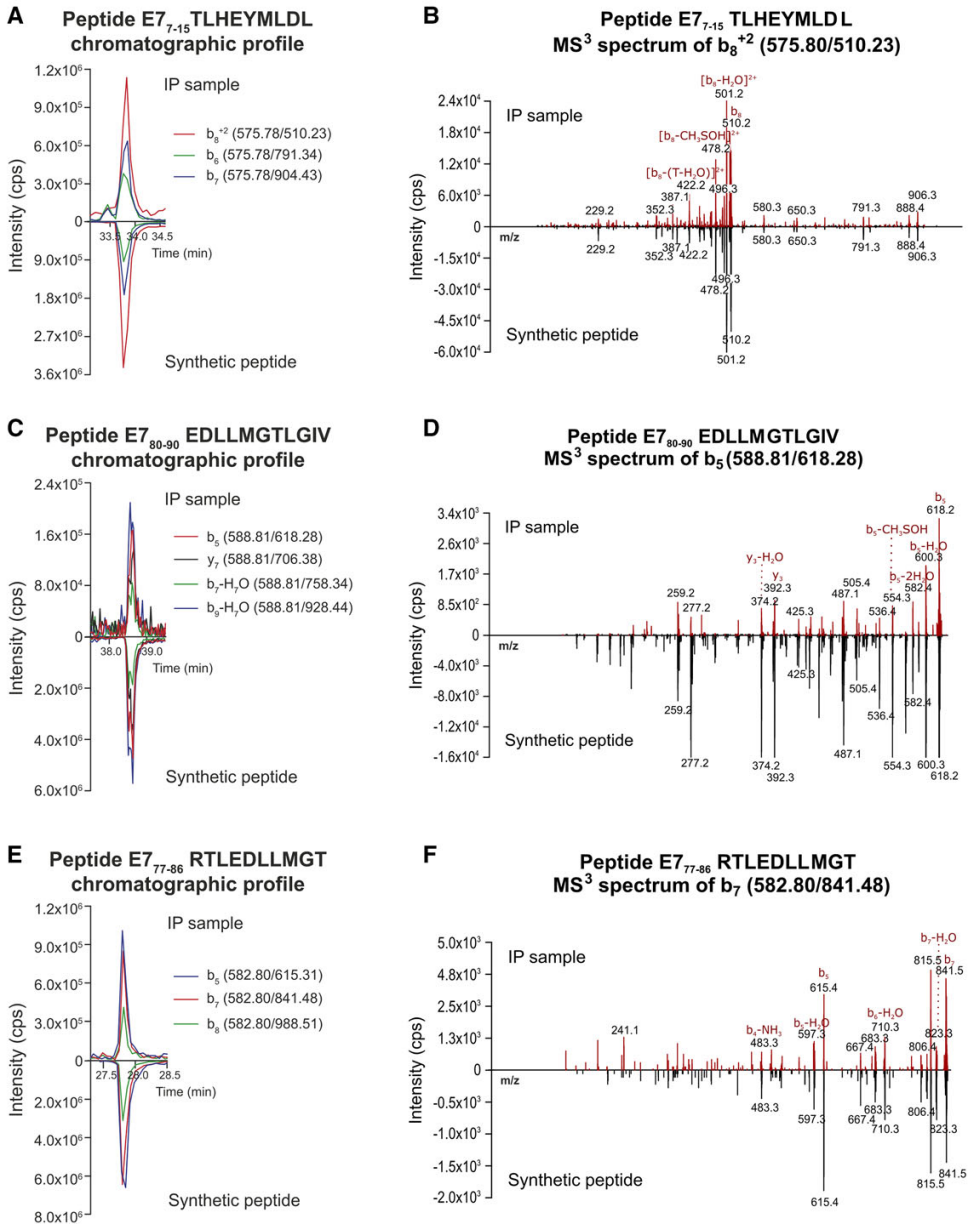
Extracted ion chromatograms for measured transitions and MS^3^ spectra for selected peptides in an IP sample and from respective synthetic peptides. A, B) Strong binder E7_7–15_ TLHEYMLDL; C, D) intermediate binder E7_80–90_ EDLLMGTLGIV; E, F) weak binder E7_77–86_ RTLEDLLMGT. CaSki IP samples were processed with Zorbax microcolumns and analyzed with LC‐MS^3^. All these Met‐containing peptides were detected in their oxidized form. For easier comparison, the results for the IP sample and the synthetic peptide are displayed on the same axis. Representative results of one out of at least three biological replicates are shown. B, D, F) *m/z* values are indicated in black, fragment annotations in red. T, threonine.

A peptide was considered to be detected when the identity criteria were fulfilled for at least three of the monitored transitions in at least two biological replicates. A peptide was considered present at the limit of detection (LOD) when only two of the monitored transitions were detected in the IP sample—but again in at least two biological replicates. The only exception is the MetOx form of peptide E7_11–19_, where the intensity of the third possible transition was so low that we excluded it from the analysis, thus only monitored two transitions, and still designated the peptide “detected” if these two transitions were seen. With this approach, we detected 11 out of the 17 monitored HPV16 peptides, three of them at LOD (Table 1). Interestingly, all detected peptides were derived from protein E7, but there was only one E6‐derived peptide among the monitored peptides from the start. Detection of a strong HLA‐A2‐binding peptide (E7_7–15_), an intermediate binder (E7_80–90_), and a peptide with low binding affinity to HLA‐A2 (E7_77–86_) are shown in Figure 3. Spectra for all other detected peptides are shown in Figure S6, Supporting Information, and details about monitored and detected transitions are given in Table S1, Supporting Information.

**Table 1:** LC‐MS^3^ detection results of HLA‐A2‐restricted HPV16 E6/E7‐derived peptides from the surface of CaSki cells

**Peptide**	**Sequence**	**MS analysis** a
E7_11–19_	YMLDLQPET	Detected
E7_11–20_	YMLDLQPETT	LOD
E7_11–21_	YMLDLQPETTD	LOD
E7_7–15_	TLHEYMLDL	Detected
E7_77–87_	RTLEDLLMGTL	Detected
E7_82–90_	LLMGTLGIV	Detected
E7_12–20_	MLDLQPETT	—
E7_11–18_	YMLDLQPE	—
E7_80–90_	EDLLMGTLGIV	Detected
E7_7–17_	TLHEYMLDLQP	—
E7_76–86_	IRTLEDLLMGT	—
E6_25–33_	ELQTTIHDI	—
E7_10–19_	EYMLDLQPET	—
E7_12–19_	MLDLQPET	LOD
E7_78–86_	TLEDLLMGT	Detected
E7_77–86_	RTLEDLLMGT	Detected
E7_81–90_	DLLMGTLGIV	Detected

a Detected: at least three monitored transitions gave MS^3^ spectrum fingerprints which matched those of the synthetic peptides. LOD, limit of detection: peptide detected with only the two most intense transitions. All detected or LOD peptides had matching retention times and extracted ion chromatogram patterns for all monitored transitions between the IP sample and the synthetic peptide, and were detected in a minimum of two biological replicates. —, not detected

Grey shading indicates HLA‐A2 binding affinity, as in Figure 2.

### 3.4 Immunogenicity Assessment of Detected Peptides

**Figure 4:**
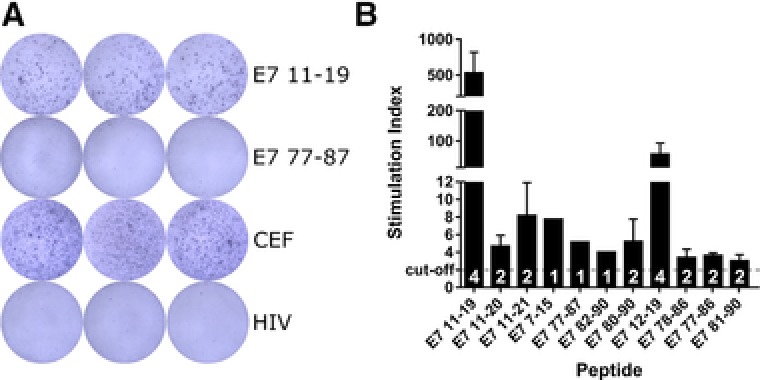
Immunogenicity assessment of detected peptides by IFN‐γ ELISpot. PBMC reactivity of 14 HLA‐A2^+^ healthy donors was evaluated by in vitro stimulation for 12 days with selected HPV16‐derived peptides. A) Representative ELISpot results of one donor showing a positive and a negative response against two HPV16‐derived peptides. CEF, positive control; HIV, negative control. B) Reactivities of all HPV16‐reactive donors (*n* = 8), shown as stimulation index (number of spot‐forming units relative to respective background control). Mean responses (±SD) across donors are shown for each peptide, cut‐off for positive responses: SI ≥ 2 (dashed line). White numbers in columns: number of reactive donors per peptide.

Confirming T‐cell reactivity against identified peptides is necessary to designate HLA‐presented peptides true T‐cell epitopes. To this end, we performed a screen for memory responses by IFN‐γ ELISpot against all 11 detected HPV16‐derived peptides with T‐cells from HLA‐A2^+^ healthy donors, which were selected for high likelihood of previous HPV encounter. Out of 14 tested donors, 8 showed reactivity against any of the tested peptides, indicating prior exposure to HPV16. Interestingly, the highest and most frequent responses were observed against E7_11–19_, which is the only peptide already detected to be presented on the cell surface of HPV16^+^ cells in a previous study.^13^ The overlapping peptide E7_12–19_ also showed responses in four donors, albeit slightly weaker than the ones against E7_11–19_. Nine more peptides elicited T‐cell responses in one to two donors (Figure 4), which means that all of the peptides detected by our targeted LS‐MS^3^ approach could be demonstrated to be immunogenic.

## 4 Discussion

To date, there are no therapeutic options for high‐risk HPV infections except surgical removal of the affected tissue. HPV‐specific immunotherapies would represent an attractive alternative. However, HPV‐derived epitopes are only presented at low abundance at the surface of infected and transformed cells, as one major HPV immune evasion mechanism is to keep the expression levels of its proteins low throughout the viral life cycle.^15^ Moreover, HPV proteins influence the antigen processing machinery and can thereby reduce cell surface presentation of certain HPV epitopes.^33^ In consequence, identification of HPV epitopes that represent valid targets for immunotherapies has so far remained a challenge.

Therefore, we here tailored a highly sensitive targeted LC‐MS^3^ approach for direct identification of naturally presented HPV epitopes. Using this strategy, we monitored 17 HLA‐A2 binders and provided evidence for the cell surface presentation of 11 HPV16 E7‐derived peptides, 10 of which were detected by MS for the first time.

LC‐MS‐based detection of HLA‐binding peptides is usually performed after IP of pHLA and subsequent peptide extraction.^34^ Most studies to date used untargeted unbiased LC‐MS^2^ detection, which detects large numbers of epitopes presented at higher abundance. Untargeted detection was used for identification of HLA binding motives,^35^ viral epitopes,^36^ and tumor mutation‐derived epitopes (neoepitopes) from cancer cell lines^37^, ^38^ or primary human tumor material.^39^, ^40^ However, this common workflow fails to identify low‐abundant peptides that might still be important for immunotherapy development. Recently, data‐independent acquisition mass spectrometry has been applied for HLA‐I peptide discovery,^41^, ^42^ which, however, needs compatible instrumentation. Another possibility that was recently utilized for sensitive detection as well as quantification of HLA‐I peptides is targeted data acquisition.^38^, ^43^, ^44^, ^45^, ^46^, ^47^ Also in the present study, we used a targeted LC‐MS^3^ approach, which is more sensitive for detection of HLA‐presented peptides than untargeted methods due to pre‐defined and pre‐programed analytes, resulting in longer acquisition times per analyte for increased sensitivity. Moreover, the optimization of the collision energy of individual transitions performed on the synthetic counterparts of the peptides to be analyzed has shown an about twofold sensitivity gain compared with the signals obtained from more generic collision energies computed from the mass of the targeted precursor, which makes this optimization process particularly suited for the detection of low‐abundant HLA‐presented peptides (reviewed in ref. 48), such as the HPV peptides investigated in this study. Furthermore, MS^3^ scanning approaches are less prone to interference of co‐eluting species than MS^1^ or MS^2^ methods.^49^, ^50^ The main downside of targeted MS^3^ approaches is a limited number of analytes that can be measured in one LC‐MS^3^ analysis. Furthermore, only a small number of dedicated bioinformatic tools is available for MS^3^ data. Therefore, a reduction of the number of target peptides is necessary. This can be achieved with in silico predictions and in vitro scanning of candidate epitopes for their experimental binding affinities. However, in vitro binding assays are laborious and are therefore often omitted. By employing predictions as well as binding assays, and analyzing only non‐cysteine‐containing peptides, we reduced the number of possible HPV16 E6/E7‐derived HLA‐A2‐binding peptides to 17 actual binders, of which 9 had never been described before (Figure 2). The binding affinity results for already reported peptides were in accordance with our results, although they were tested with different in vitro assays.^12^, ^13^

We adapted the isolation of pHLA from typical on‐column to in‐solution IP to reduce volumes and contact surfaces for minimal losses of low‐abundant target peptides, as it has been shown that the IP step introduces most losses during sample pretreatment.^46^ Moreover, our approach does not require peristaltic pumps or special LC equipment and can therefore be performed in any laboratory. For low‐abundant peptides, maximal recovery and high preparative yields are particularly relevant throughout the whole peptide purification pipeline. Thus, we exchanged the commonly used ultrafiltration with RP material extraction. We compared three RP materials; OligoR3, Zorbax and Sep‐Pak. The best target peptide recoveries and the most effective removal of protein contaminants were achieved with Zorbax microcolumns. Thus, this material was chosen for our standard HLA‐I peptide purification workflow. The high performance of the Zorbax material within this application may be explained with its pore size. According to the product specifications, the pore sizes were 80 Å, 130 Å, and 300–3000 Å for Zorbax, Sep‐Pak, and OligoR3, respectively. The bigger the pore size, the more proteins can bind to the RP material. This underlines that the pore size of RP material is an important parameter to be considered for separation of proteins and peptides^51^, ^52^ and can be exploited for HLA‐I peptide isolation.

Cell lysis for pHLA IP is usually performed with lysis buffers containing non‐denaturing detergents. The most common detergents are CHAPS and IGEPAL CA‐630. Detergents have to be removed before downstream LC‐MS analysis as they significantly influence peptide separation on the LC column^53^, ^54^ or interfere with ionization of analytes.^55^, ^56^ Therefore, thorough and repeated washing of IP samples is necessary. If for reasons inherent to experimental setups detergent removal from the IP sample cannot be performed, we demonstrated that non‐ionic detergents (such as IGEPAL CA‐630) could be removed by ion exchange (IXC) during the subsequent peptide extraction. This is not possible for zwitterionic detergents such as CHAPS. The choice of IXC material depends on the sequence of target peptides, which is tightly connected to the HLA binding motives and is distinct for each HLA allele.^3^, ^4^ For example, the anchor residues on position 9 (P9) in HLA‐A11‐restricted peptides are typically R or K, which results in peptides with at least one basic amino acid, whereas the preferential amino acids for HLA‐A2‐binding peptides on P2 and P9 are small or aliphatic hydrophobic residues (L, I, V, M, A, T, or Q).^3^ As another example, Met is often found on P1 and P3 in HLA‐A2‐binding peptides. This results in many hydrophobic, Met‐containing peptides, often without any basic amino acid, which was also reflected in the characteristics of the target peptides in this study. As the SCX Poros 20HS material, which gave the best results for our HPV16‐derived HLA‐A2‐restricted peptides, is known to work best with basic peptides, it is reasonable to assume that it will also be suited for other HLA peptides with more basic chemical characteristics.

Our optimized peptide isolation strategy and highly sensitive targeted LC‐MS^3^ analysis resulted in identification of 11 HLA‐A2‐restricted HPV16 E7‐derived peptides on CaSki cells (Table 1) out of 17 monitored. The reason for this rather high ratio may be our strategy of only looking for peptides whose HLA‐A2 binding capacity had been experimentally confirmed, which is in contrast to most other studies, which search for predicted HLA‐binding peptides. Only one identified peptide, E7_11–19_, was detected by MS before.^13^, ^14^ The major difference of our strategy compared to the approach described in ref. 13, 14 is a faster and more sensitive MS instrumentation. This allowed for online LC‐MS^3^ analysis of IP samples and measurement of more peptides in one analysis, whereas the authors of the earlier studies measured only one peptide at a time via static nanospray.

Interestingly, many detected peptides were predicted as HLA‐A2 non‐binders with the 500 nm cut‐off recommended by the prediction algorithms, indicating that this cut‐off may be too strict. This phenomenon was also observed for HLA‐B*3503 binding peptides identified by MS, which had been predicted as non‐binders.^39^ Furthermore, we detected peptides from the whole range of experimental binding affinities, half of them intermediate or low‐affinity binders. This shows that it is important to not only focus on the best (predicted) binders, but to also monitor peptides with moderate or weak binding affinities to the respective HLA molecule, as these also may be presented to T‐cells for immune recognition. Indeed, we found T‐cell reactivity against all of the 11 MS‐detected peptides (Figure 4), suggesting that our detection approach has high predictive value for immunogenicity. Obviously, this will have to be tested in larger data sets, but stresses the importance of correlating MS‐verified HLA‐presented peptides and immunogenicity data, an issue that is currently not addressed by the existing literature (reviewed in ref. 57).

In conclusion, we here describe a highly sensitive targeted LC‐MS^3^ methodology for detection of low‐abundant epitopes on the surface of cancer cells. We are currently applying this methodology to HPV peptides presented by other HLA molecules, and are working on methods allowing monitoring of Cys‐containing peptides. These results will contribute to a better understanding of HPV‐mediated changes in epitope presentation, and provide a solid basis of targets for HPV immunotherapy development. As the sequences of possible mutation‐derived tumor neoepitopes are known from tumor genome sequencing projects, our methodology can also be used for ascertaining the presentation of neoepitopes on the tumor cells surface. This in turn will facilitate the development of immunotherapies for other tumor types. On a broader scale, knowledge about truly presented epitopes will improve epitope prediction servers, which will hopefully make target epitope definition an easier task in the future.

## Conflict of Interest

The authors declare no conflict of interest.

## Supporting information

Supporting information.

Supporting information.
