# Supplementary material for: A Targeted LC‐MS Strategy for Low‐Abundant HLA Class‐I‐Presented Peptide Detection Identifies Novel Human Papillomavirus T‐Cell Epitopes
Source: Proteomics. 2018 May 2;18(11):1700390. doi: 10.1002/pmic.201700390 (PMC6033010; doi:10.1002/pmic.201700390)
Supplement: Supplementary file 1 — Supporting information. [file PMIC-18-na-s001.docx]

**Supplementary Materials and Methods**

**1) Assessment of different peptide purification approaches**

To separate peptides from HLA molecules and other protein contaminants contained in an IP sample, we compared various ultrafiltration and reverse phase approaches. The resulting protein fractions were subjected to in-solution reduction, alkylation and digestion, and subsequently analyzed by LC-MS with solid-phase extraction capillary liquid chromatography coupled to a Q-Exactive instrument. Data analysis was performed by Skyline and MaxQuant.

- 1. **Purification of peptides from IP samples by ultrafiltration and reverse phase materials**

The ultrafiltration devices (Vivacon 500 2 kDa or 10 kDa cut-off, Sartorius Stedim, Göttingen, Germany; Amicon 3 kDa cut-off, Merck-Millipore, Cork, Ireland) were washed with 50% methanol (MeOH; Biosolve, CE Valkenswaard, The Netherlands) in water before sample ultrafiltration, and again afterwards to recover remaining peptides. The flow-through (FT) from sample and wash fractions contained peptides and were collected together. They were concentrated with vacuum centrifugation.

Peptide RP purification was performed with 1 mL Sep-Pak tC18 cartridges (Sep-Pak; Waters, Milford, MA, USA),with in-house assembled microcolumns in 200 µL pipette tips as in ^[1]^, filled with Zorbax SB-C18 5 µm material (Agilent Technologies, Santa Clara, CA, USA) or with OligoR3 50 µm (Applied Biosystems, Bedford, MA, USA). Samples were bound, washed with 0.1% TFA and eluted with 30% ACN (Biosolve) in 0.1% TFA if not otherwise specified.

**1.2) In-solution reduction, alkylation, digestion**

Dried protein fractions after gradual elution of IP samples from RP materials were resuspended in
40 µL 50% 2,2,2-trifluoroethanol (Fluka Analytical, Schnelldorf, Germany) in water, incubated 1h at 65ºC with 300 rpm mixing. Subsequently, 3.5 µL of 100mM DTT (Sigma-Aldrich, Taufkirchen, Germany) in 25 mM ammonium bicarbonate were added and incubated for 30 min at 65º C with 300 rpm mixing. 5 µL of 200 mM iodoacetamide (IAA; Sigma-Aldrich) in 25 mM ammonium bicarbonate were added, followed by incubation for 30 min at 65º C in the dark with agitation (300 rpm). 180 µL of 25 mM ammonium bicarbonate and 0.2 µg of trypsin (Sigma-Aldrich) were added to the sample and incubated overnight at 37º C with agitation (300 rpm). The reaction was quenched by addition of 10% TFA in water for the final concentration of 1% TFA.

**1.3) LC-MS measurements**

Samples were analyzed with solid-phase extraction capillary liquid chromatography (speLC) directly coupled to a Q-Exactive instrument (Thermo-Fischer, Bremen, Germany) as in ^[2]^. Briefly, samples were loaded onto disposable micropipette solid phase extraction C18 tips (StageTips, Thermo Fischer Scientific) which allowed for direct concentration and desalting of samples. Samples were eluted from the StageTip resin during the LC gradient and separated on 7 cm in-house produced analytical columns (inner diameter 200 µm; Polymicro Technologies, Molex CM Scientific, Silsden, UK) packed with 3 µm ReprosilPur-AQ 120 C18 material (Dr. Maisch, Ammerbuch-Entringen, Germany). Solvent A was 0.1% formic acid (FA) in H_2_O (Fluka Analytical) and solvent B was composed of 0.1% FA (Sigma-Aldrich) in 100% ACN (Fluka Analytical). Separation started with an initial 1 min equilibration of the column with 4% solvent B, followed by a linear gradient to 35% solvent B in 5 min. The flow rate was 1.5 µL/min.

The Q Exactive instrument was operated in the full scan Top2 data dependent acquisition mode. The method utilized mass resolutions of 35000 and 17500 for full-scan and MS^2^ measurements, respectively. Mass ranges were set to 400-1600 m/z, fill times were set to 50 ms for full scan and to 100 ms for MS^2^. Automatic gain control (AGC) target values were set to 3x10^6^ for full scan and to 5x10^5^ for MS^2^.

**1.4) MS^1^ quantification with the Skyline software**

Quantification of HLA-A2-restricted HPV16 E6 and E7-derived peptide recovery after ultrafiltration and RP processing was performed with the Skyline program^[3,^ ^4]^ on the MS^1^ level for target peptides in Met and MetOx form. Peak selection criteria were retention time, mass tolerance 4 ppm, isotopic distribution of peptide precursor and concurrent signals for peptides in different charge states. Quantification results were visualized with the Prism 6 software (GraphPad, LaJolla, CA, USA) (Supplementary Figure 1).

#### 1.5) LC-MS^2^ data processing, protein identification and relative quantification with MaxQuant

Raw data of speLC-Q Exactive-analyzed protein fractions eluted from RP materials was searched against the human and mouse sequences from the UniProt database (retrieved December 10, 2014) with the MaxQuant 1.5.2.8 software.^[5]^ The mouse sequences were used to identify anti-HLA-A2 antibody derived peptides, whereas the human sequences were applied for the identification of other cell components. Identified peptides were matched across different samples and LC-MS replicates. Database searches were performed with trypsin as the digestion enzyme with one missed cleavage allowed, carbamidomethyl as fixed modification on Cys, and oxidation on Met as variable modification and with 1% FDR. Intensities of unique peptides and a MaxQuant score ≥60 were used for relative quantification. Protein quantification was performed by summing peptide intensities of ≥3 unique peptides per protein and results visualized with the Prism 6 software (Supplementary Figure 2).

**2) Assessment of detergent removal**

To examine the removal of the nonionic detergent IGEPAL CA-630 from IP samples with ion exchange (IXC), we measured peptide recovery and detergent removal efficiency. First, different strong cation exchange (SCX) and strong anion exchange (SAX) exchange materials were compared to test target peptide recovery. This was analyzed by targeted LC-MS^2^. Subsequently, the efficiency of IGEPAL CA-630 removal by the material giving the highest target peptide yield (SCX Poros 20HS microcolumns) was assessed by analyzing the resulting fractions by MALDI-TOF MS^1^.

**2.1) Detergent removal**

IXC was performed with microcolumns filled with SCX Poros 20HS, SCX Poros 20S, SAX Poros 20HQ (all Applied Biosystems) or SAX Poros 50XQ (Thermo Fischer). SCX binding and wash buffer contained 20 mM maleic acid in water, pH=1.3 (adjusted with HCl). SCX elution buffer contained
50 mM piperazine, 30% ACN, 1 M NaCl in water, pH~11. SAX binding and washing buffer contained 20 mM piperazine in water, pH=10.9. SAX elution buffer contained 50 mM maleic acid, 30% ACN, 1 M NaCl in water, pH=1.4 (adjusted with HCl). Eluted samples were concentrated by vacuum centrifugation to a volume of 30 µL and desalted using Zorbax microcolumns before LC-MS analysis.

**2.2) Targeted LC-MS^2^ measurement**

To generate a targeted LC-MS^2^ spectra library, synthetic peptides were analyzed first with untargeted acquisition on a nanoAcquity UPLC (Waters) coupled via a NanoSpray III ESI ion source to a QTrap6500 (both Sciex, Foster City, CA, USA) (nanoAcquity UPLC-QTrap6500) instrument. Peptides were loaded on a BEH C18 25 cm x 75 µm column with 1.7 µm particle size (Waters) and separated via a steep linear gradient with a flow rate of 300 nL/min from 3% B to 10% B in the first minute, followed by a slower linear gradient from 10% B to 40% B until minute 50. The LC-MS grade mobile phases were composed of 0.1% FA, 0.01% TFA (ProteoChem, Denver, CO, USA) in water (Biosolve) (phase A) and of 0.1% FA, 0.01% TFA in ACN (Biosolve) (phase B).

The MS instrument was operated in low mass profile with the NanoSpray III ESI ion source operated at 3000 V. Curtain gas was set to 30 psi, ion source gas to 15 psi, collision gas (CAD) was set to “high” and interface heater temperature was 150° C. Acquisition occurred in Top3 data dependent mode with enhanced mass spectrum full scan and the following IDA selection criteria: ions with a charge of +2 or +3 were confirmed with enhanced resolution scan, m/z range was 300-1000, rolling collision energy was enabled, former target ions were excluded after 5 occurrences for 30 s, as well as isotopes within 4 Da. Mass tolerance was set to 0.25 Da. MS^2^ spectra were acquired in enhanced product ion mode using quadrupole (q2) fragmentation, low Q1 resolution, scan speed of 10000 Da/s and m/z range of 250-1000.

**2.3) Data analysis with ProteinPilot, Paragon and Skyline**

ProteinPilot 4.5 (Sciex) data base search was used to assign peptide sequences to MS^2^ spectra. For the respective MRM method building, we included the HLA-A2-restricted HPV16 E6 and E7-derived target peptide sequences and a yeast database (retrieved from UniProt on July 21, 2016) as a decoy reference. This was done for statistical reasons, as too small a search space might impact the false discovery rate (FDR). The search was performed with the Paragon algorithm.^[6]^ Sample type was set to identification; digestion enzyme, species and Cys alkylation were set to none, instrument was set to QTrap6500 ESI. The search effort was set to thorough ID and FDR to 1%. ProteinPilot 4.5 search results were used for spectral library generation in the Skyline software.^[3, 4]^ Optimal three to six MRM transitions were selected and measuring conditions (collision energy) for each synthetic peptide were optimized. For Met-containing peptides, optimization was performed with oxidized (MetOx) and non-oxidized (Met) peptide. When assignment of MRM transitions was not possible for each peptide and its MetOx counterpart with the Skyline software, we added them manually based on already optimized parameters of the counterpart. MRM acquisition was performed with Q1 and Q3 operated at unit resolution and dwell time 25 ms for each transition. For MRM quantification, we adhered to the following rules: RT was required to be at expected time, all MRM transitions were required to be measured concurrently and in correct hierarchy of abundance. Data were visualized and analyzed manually with Skyline. The manually added transitions were quantified with manual peak integration with Analyst software 1.6.2 (Sciex). Quantification results were visualized with Prism 6 software (Supplementary Figure 3).

**2.4) MALDI-TOF MS^1^ measurement**

Fractions generated during IGEPAL CA-630 removal with SCX Poros 20HS were diluted 1:100 in 50% ACN (Biosolve)/0.1% TFA (ProteoChem), mixed 1:1 (v/v) with 3 mg/mL CHCA (ProteoChem) solution in 0.1% TFA in 80% ACN and spotted on MALDI target plates in triplicates. Measurements were conducted with a MALDI TOF/TOF 5800 (Sciex) and data analysis was performed with TOF/TOF Explorer Software V4.1.0 (Sciex) as described in ^[7]^. Briefly, MS^1^ spectra acquisition was performed in positive reflector mode with 1000 shots per spectrum. Mass range was set to 750 to 4000 Da with a focus mass of 2000 Da. The sample plate was moved with continuous stage motion with a stage velocity of 1000 µm/s during acquisition. Laser input bandwidth was 1000 MHz and pulse rate was 400 Hz. The MS^1^ spectra processing method used the following peak detection characteristics: minimal S/N was set to 5, local noise window width to 250 m/z and minimal full peak width at the half maximum to 1 bin. Raw spectrum filtering contained fast Fourier transformation peak smoothing and Poisson denoising. Cluster area S/N optimization was performed with a S/N threshold of 20. Four peptides (TVFDEAIR, MW=951.06; TGVFDEAIRTVGF, MW=1411.72; CLEHMYHDLGLVRDF, MW=1846.87 and EEQPSTPAPKVEQQEEILC, MW=2155.02) were spiked into the sample matrix and were used as reference for internal calibration. Only monoisotopic peaks were used and at least one peak was required to match. 0.3 m/z were taken as mass tolerance; maximal outlier error was set to 10 ppm. Results were again visualized with Prism 6 software (Supplementary Figure 4).

**3) Targeted LC-MS^3^ measurement for detection of peptides presented by HLA molecules**

If not stated otherwise, targeted LC-MS^3^ measurements were performed with the same LC separation and nano-ESI parameters as described above for “2.2) Targeted LC-MS^2^ measurement” on the nanoAcquity UPLC-QTrap6500 instrument. Parameters for MS^3^ measurement were optimized by manual injection of synthetic peptides into the QTrap6500. Synthetic peptides were diluted to a final concentration of 0.5-2.0 µg/mL in 0.1% FA in 50% ACN and injected into the QTrap6500 instrument with the built-in syringe pump maintaining a constant flow of 5-10 µL/min. The positive electron spray voltage on the TurboV ion source was set to 5500 V. The curtain gas was set at 30 psi, ion source gas at 15 psi, CAD high and interface heater temperature at 150° C. The instrument operated in the high mass hardware profile. Q1 resolution was set at unit resolution and the linear ion trap (LIT) was set to LIT resolution with 1000 Da/s scan.

Collision energy (CE) and declustering potential (DP) were optimized for the best signal-to-noise ratio using the compound optimization script of the Analyst 1.6.2 software for the 10 most intense MS^2^ fragments with m/z 400-1000 Da. The average DP and CE values of three optimization replicates were used to optimize the excitation potential (AF2), which was performed with ramping from 0.0 to 0.2 V. Optimal AF2 for MS^3^ generation was determined when the MS^2^ precursor/fragment intensity was reduced to 5% of its starting intensity. DP, CE and AF2 parameters of a minimum of three fragments of ≥ 5 amino acids per peptide (except for the MetOx form of E7_11-19_ with only two fragments) were used for LC-MS^3^ method generation and determination of the retention time for scheduling. MS^3^ parameters optimized for Met-containing peptides were used also for measuring of MetOx counterparts, with precursor and fragment ion masses increased by 16 Da.

The identity of target peptides in the IP sample was confirmed by manual comparison of LC-MS^3^ results of synthetic peptide and IP sample results. The criteria for positive identification were defined as outlined by ^[8]^: matching retention times, matching extracted ion chromatograms for the monitored transitions (concurrence and hierarchy of abundance) and matching MS^3^ fingerprints between the synthetic peptide and the peptide identified in the IP sample.

The optimized parameters for target peptides are listed in Supplementary Table 1. Data have been deposited in PeptideAtlas, with the Identifier PASS01152. Besides HLA-A2-restricted HPV16 E6 and E7-derived peptides, endogenous peptides AIVDKVPSV from coatomer subunit gamma-1 and YLLPAIVHI from ATP-dependent RNA helicase DDX5^[9]^ were monitored in each IP sample to verify successful HLA-A2 IP. Chromatograms and MS^3^ spectra were visualized with Prism 6 software (Figure 3 and Supplementary Figures 5 and 6).

**Supplementary Results and Figures**

***1) Optimization of the sample preparation workflow***

*1.1) Comparison of ultrafiltration with reverse phase (RP) purification*

As our HPV target peptides are known to be of low abundance from the start (due to low level expression of the viral proteins from which they derive), we aimed to optimize the sample preparation workflow for minimal peptide losses. In most HLAp isolation workflows, an ultrafiltration step is performed after IP. However, high peptide losses can be caused by ultrafiltration. Thus, we compared three ultrafiltration devices (Vivacon with two cut-offs, Amicon) with two RP materials (Seppak, Zorbax) for optimal peptide extraction. Overall, RP materials (especially Zorbax) showed markedly higher yields of our target peptides (Supplementary Figure 1).


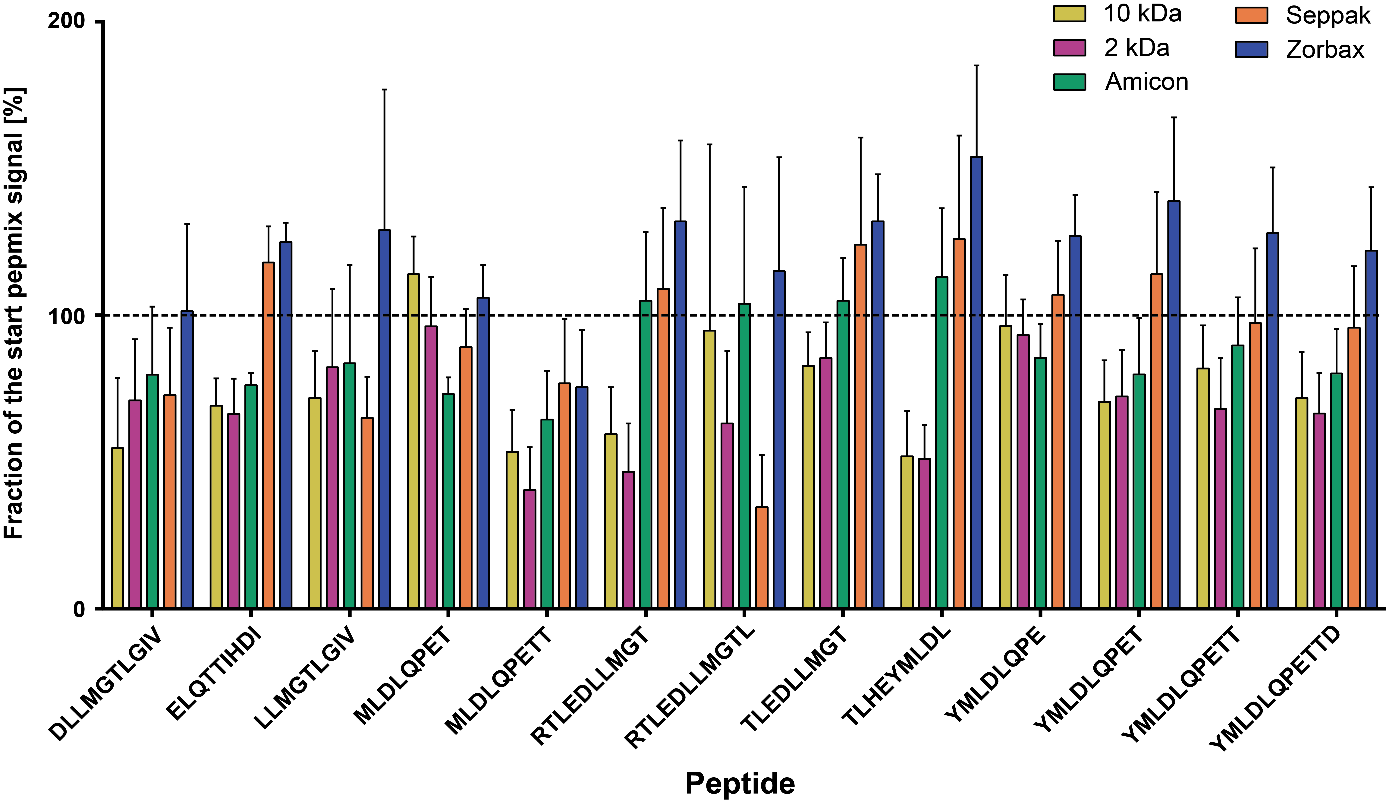


Supplementary Figure 1. Comparison of different epitope extraction methods from an IP eluate.

A mixture of HLA-A2-restricted HPV16 E6 and E7-derived synthetic peptides was spiked into an HLA-A2 IP sample from BSM HPV16-negative cells to ensure the same starting conditions for each extraction method. Samples were processed, vacuum dried and analyzed with the speLC-Q-Exactive instrument. Data processing and MS^1^ relative quantification were performed with Skyline. Peptide intensities in every sample were compared to the intensities in the unprocessed starting peptide mixture. Signal intensities of Met-containing peptides were calculated as intensity sum of oxidized and non-oxidized peptides. 10 kDa: ultrafiltration with 10 kDa Vivacon ultrafilters, 2 kDa: ultrafiltration with 2 kDa Vivacon ultrafilters, Amicon: ultrafiltration with 3 kDa Amicon ultrafilters, Seppak: processing with the Sep-Pak cartridge, Zorbax: processing with the Zorbax microcolumn. Error bars represent SD from three technical replicates.

*1.2) Comparison of different RP materials*

Next, we tested protein separation from peptides by differential elution from RP materials. Ideally, peptides bind to the RP material, while contaminating proteins are discarded in the flow through (FT). Three RP materials (Sep-Pak, Zorbax and OligoR3) were examined. We observed that even small amounts of IP samples clogged OligoR3 microcolumns, while this was not the case for Sep-Pak cartridges and Zorbax microcolumns. Analysis of protein content revealed that the FT fraction of Zorbax micro-columns and Sep-Pak cartridges contained most of the contaminating proteins contained in the IP sample, which is demonstrated by overlapping chromatographic profiles of unprocessed IP sample and the respective FT fractions (purple and dark blue in Supplementary Figure 2A, B). This indicates successful separation of peptides from proteins. On the contrary, the FT fraction of OligoR3 microcolumns contained minimal protein amounts (data points to the very right in Supplementary Figure 2C), indicating that most of the proteins were bound to the OligoR3 material and thereby caused the clogging. Due to the clogging, only the initial FT fraction could be analyzed, but no wash or elution fractions as for the other materials.

**
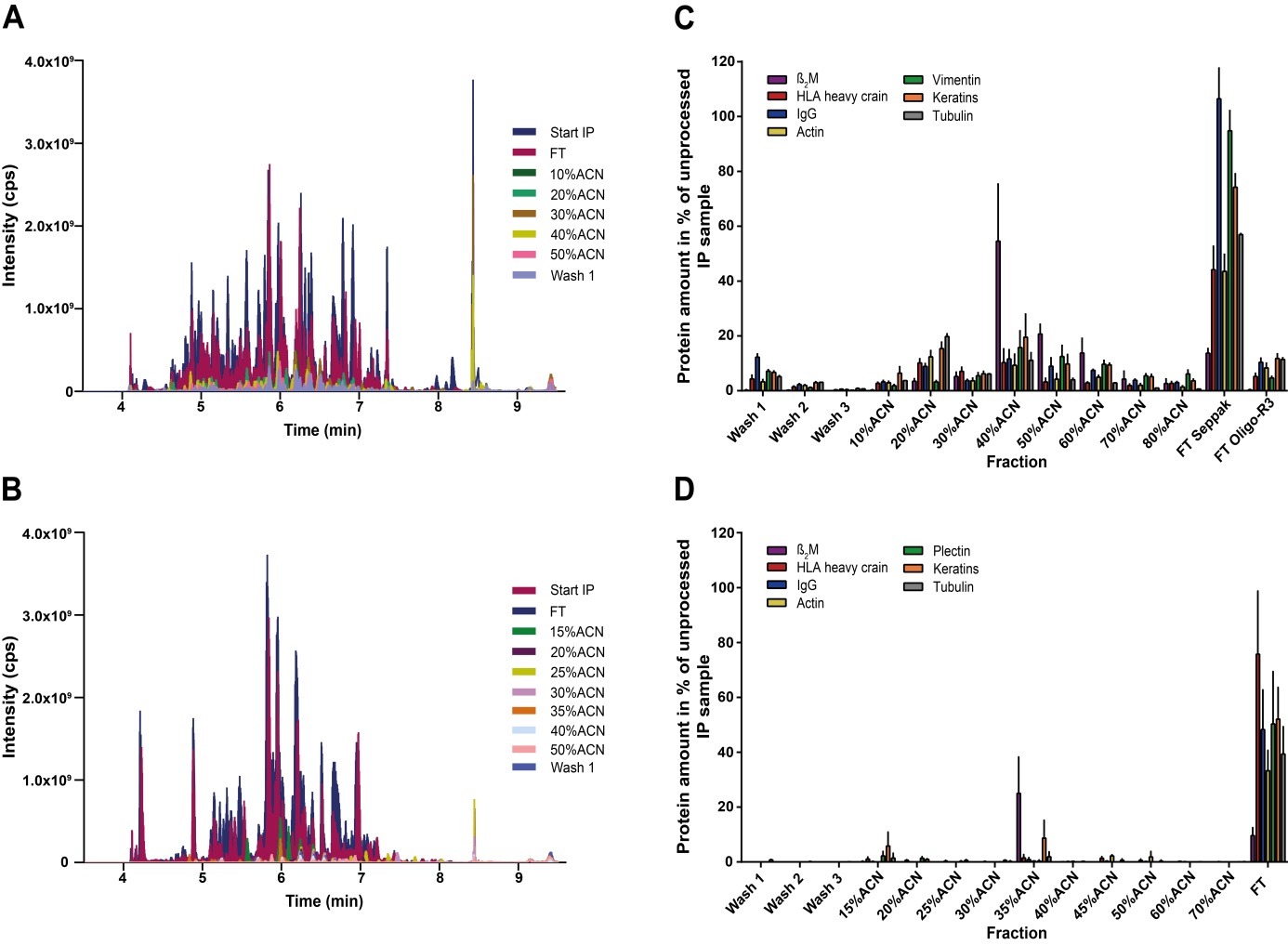
**

**Supplementary Figure 2. Comparison of different RP materials.**

Chromatographic profiles (A, B) and relative protein quantification (C, D) of IP samples after fractionation on Sep-Pak cartridges (A, C), Zorbax microcolumns (B, D) or OligoR3 microcolumns (C, data points to the very right). IP samples were bound to the respective RP material and gradually eluted with solvents containing low to high % ACN. Fractions were collected and subjected to in-solution reduction, alkylation and digestion, and analyzed on the speLC-Q-Exactive platform. Protein quantification was performed with MaxQuant. Protein intensity in a given fraction is relative to the intensities of an unprocessed IP sample. FT: flow-through fraction, % ACN: fractions eluted with X% ACN/0.1% TFA, Wash1: first wash after IP sample binding. Quantified proteins are indicated. IgG: sum of intensities for heavy and light antibody chains, actin and tubulin: sum of intensities for alpha and beta protein, keratins: sum of intensities for I-14, I-17, II-6 and II-7 keratins. Mean±SD from a minimum of two technical replicates is displayed.

Relative quantification confirmed that the majority of IP proteins did not bind to the Sep-Pak and Zorbax materials (Supplementary Figure 2C, D), indicating successful separation from peptides. However the smallest protein contained in IP samples, β_2_M (11 kDa), bound to both materials and eluted at >30% ACN. As Zorbax microcolumn processing resulted in overall lower final protein amounts and best target peptide recoveries, we chose it as our standard epitope extraction strategy.

*1.3) Detergent removal*

Since high detergent content in the sample can interfere with downstream LC-MS analysis, it needs to be removed either directly after the pHLA IP by extensive washing or during peptide extraction from acidified IP samples. To examine the removal of the nonionic detergent IGEPAL CA-630 from IP samples with ion exchange (IXC), we measured peptide recovery and detergent removal efficiency in separate experiments.

Different strong cation exchange (SCX) and strong anion exchange (SAX) materials were compared to test target peptide recovery. IXC conditions were set to allow for both, highest possible binding of the target HPV16 peptides to the resin, and efficient detergent removal. Thus, binding and wash buffers had the same composition with minimal ion strength and low pH (~1.1) for SCX or high pH (~11) for SAX. The results of IXC processing showed target peptide losses, which were smaller for SCX than for SAX materials. We chose the IGEPAL CA-630 removal strategy with the SCX material Poros 20HS for our workflow since it showed highest peptide yields (Supplementary Figure 3).


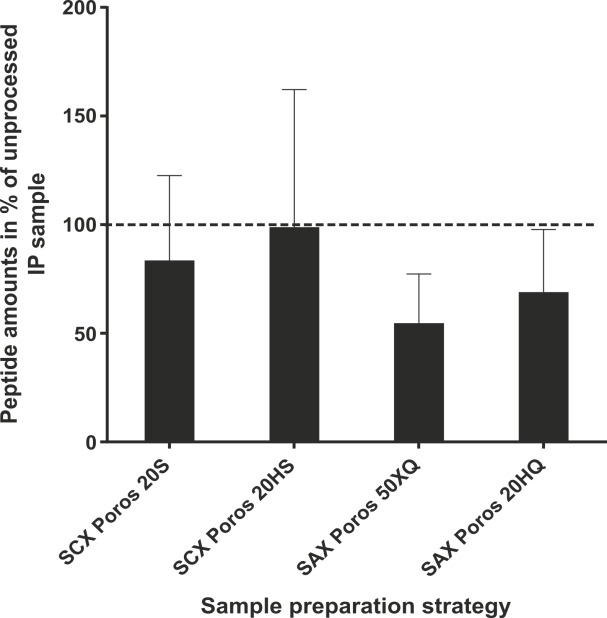


**Supplementary Figure 3. Peptide yields after processing with different IXC materials.**

HPV target peptides were added into an IP sample from HPV16-negative BSM cells. Equal amounts of sample were purified through each of IXC materials, desalted and measured with targeted LC-MS^2^. Signals were first normalized to the spike-in peptides and second to the unprocessed sample. Total peptide mean intensity±SD was calculated from at least 2 experimental replicates.

Next, we examined the efficiency of IGEPAL CA-630 removal on the SCX Poros 20HS micro-columns (Supplementary Figure 4). The unprocessed sample and FT fraction contained the most intense detergent signals (Panels A and B, respectively), whereas the SCX Poros 20HS microcolumn wash fraction and eluate contained no or minimal detergent amounts (Panels C and D).


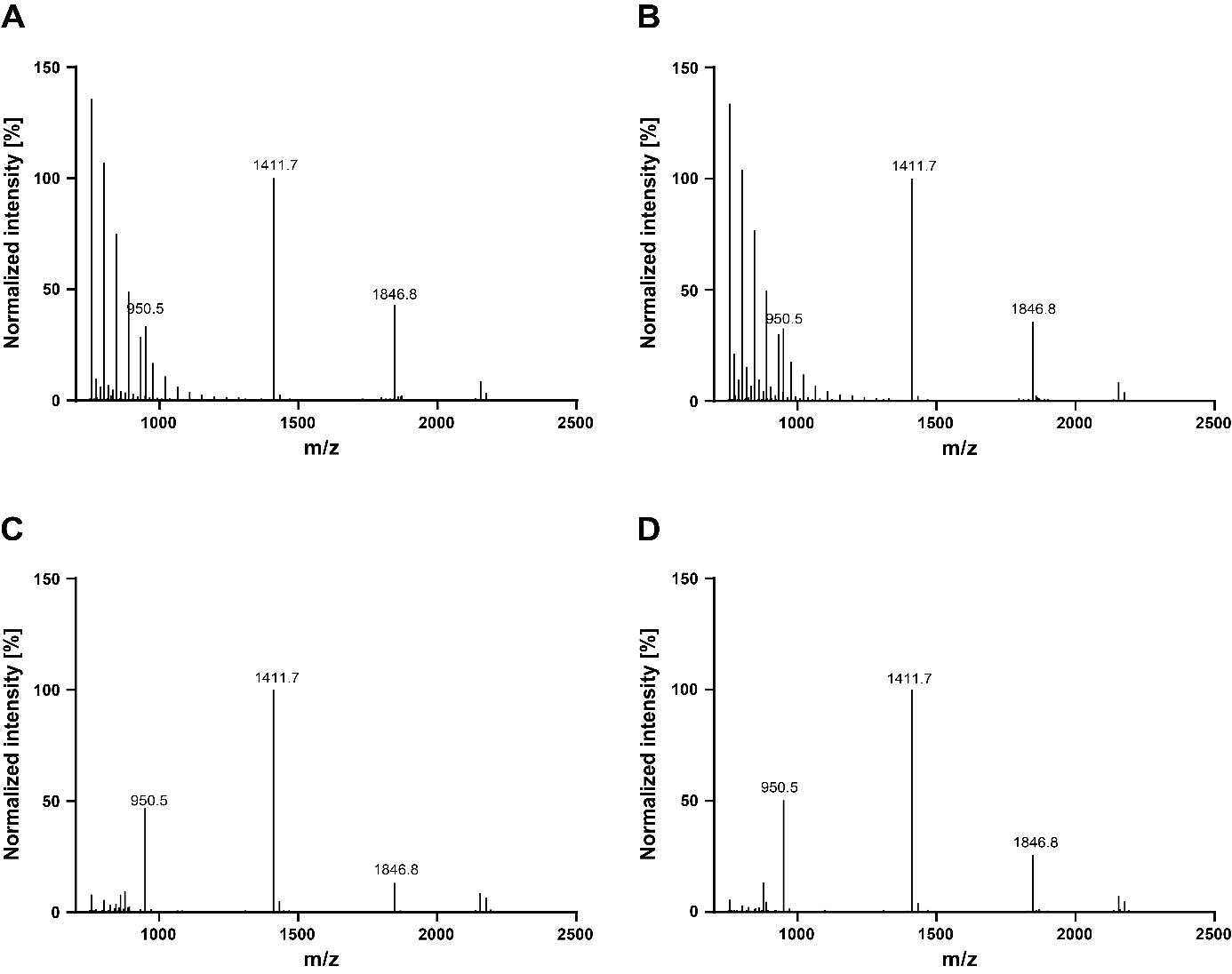


**Supplementary Figure 4. Removal of IGEPAL CA-630 with IXC.**

IGEPAL CA-630 was added into the SCX binding buffer and processed with SCX Poros 20HS micro-columns in two experimental replicates. Fractions were collected, diluted and analyzed with reflector mode MALDI TOF MS^1^ in three technical replicates. MS^1^ spectra normalized to the spike-in peptide TGVFDEAIRTVGF (MW=1411.7) for unprocessed sample (A), SCX flow through (B), SCX wash (C) and SCX eluate (D) are displayed for a representative experiment. Intense detergent signals are between 700 and 1100 m/z with a characteristic m/z difference of 44, which corresponds to the IGEPAL CA-630 repeat unit.

Taken together, processing through SCX Poros 20HS micro-columns removed excess IGEPAL CA-630. However, SCX processing resulted in peptide losses. Thus, extensive washing of the IP sample directly after IP remains the preferred method for detergent removal.

***2) Detection of HLA-A2 presented peptides by targeted LC-MS^3^ mass spectrometry***

*2.1) LC-MS^3^ detection challenges of methionine-containing peptides*

Out of 17 HLA-A2-restricted HPV16 E6 and E7-derived target peptides 16 contained methionine (Met), prone to oxidation during sample purification or mass spectrometric analysis, which results in methionine sulfoxide (MetOx). Therefore, we monitored oxidized and non-oxidized peptides with targeted LC-MS^3^ analysis. The LC separation of peptides without MetOx resulted in narrow chromatographic peaks (Supplementary Figure 5, upper panels). However, the LC chromatographic profiles of MetOx-containing peptides (lower panels) in a simple synthetic peptide mixture (lower panel A) or spiked into an E. coli digest as irrelevant complex background (lower panel B) resulted in long peak broadening on the BEH C18 column. The phenomenon of peak broadening was observed for every measured MetOx-containing peptide. Interestingly, peak broadening of MetOx containing peptides was not present in IP samples (lower panel C), suggesting that the IP sample matrix has a positive effect on the separation of MetOx peptides on BEH C18 columns.


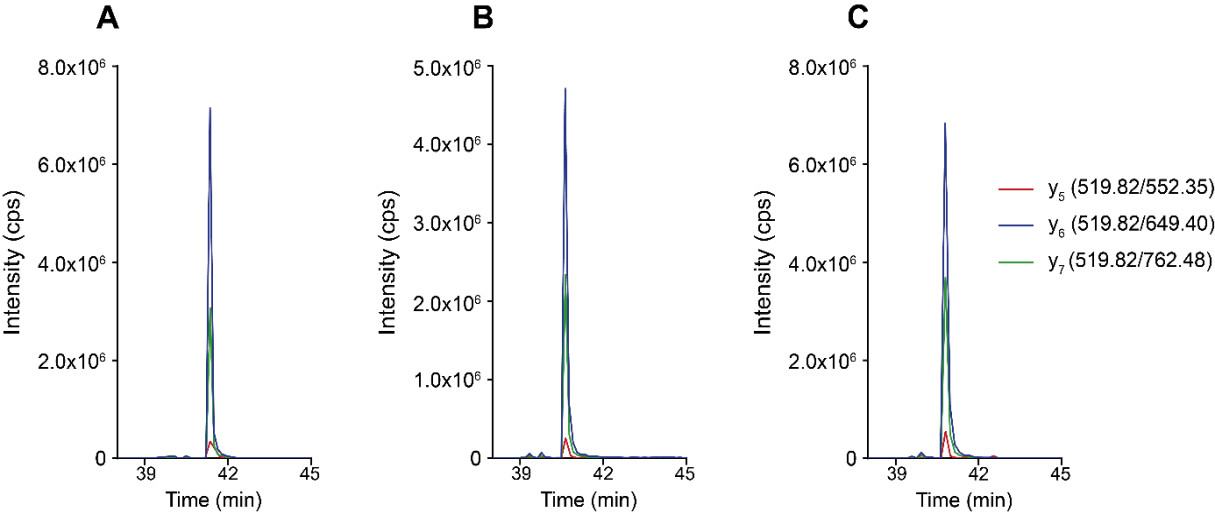

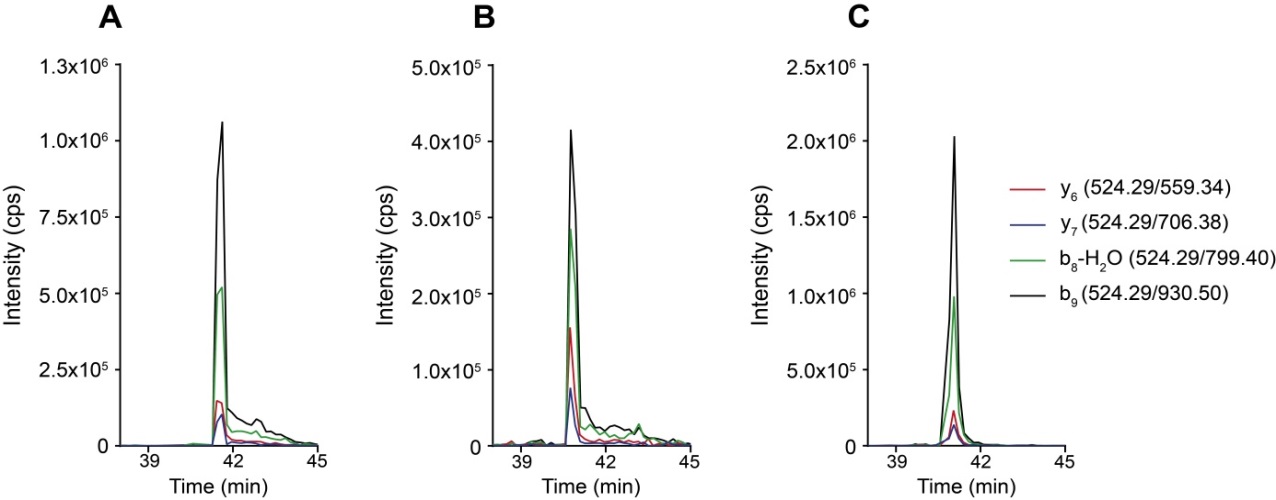


**Supplementary Figure 5. Peak shapes of a non-Met-containing and of a MetOx-containing peptide on the BEH C18 column.** Upper panels: endogenous (non-Met-containing) HLA-A2-restricted control peptide YLLPAIVHI. Lower panels: HLA-A2-restricted HPV16-derived MetOx-containing peptide E7_81-90_ DLLMGTLGIV. (A) Pure peptide mixture of HLA-A2-restricted HPV16 E6 and E7-derived and endogenous control peptides. (B) Peptides added into 80 ng of E. coli lysate digest or (C) added into a Zorbax microcolumn-desalted HLA-A2 IP sample from HPV16-negative BSM cells. All samples were analyzed with LC-MS^3^. Overlaid extracted ion chromatograms for monitored MS^3^ transitions are displayed.

*2.2) Identification of HLA-A2-presented HPV16-derived peptides*

All 17 HPV16-derived peptides that were determined to be HLA-A2 binders in the cellular binding assays and 2 endogenous control HLA-A2 binders (derived from housekeeping proteins) were monitored by targeted LC-MS^3^ in CaSki IP samples prepared with our optimized sample preparation protocol. The control peptides were required to be detected to verify successful HLA-A2 IP, and indeed were found in all samples. A minimum of three biological replicates was measured and a minimum of three transitions was monitored for each peptide. The identity of target peptides in IP samples was confirmed with several criteria, as outlined in the Methods section. All detected transitions are indicated in Supplementary Table 1. A peptide was designated “detected” when at least three of the monitored transitions were seen in at least two biological replicates. A peptide was considered to be detected at the limit of detection (“LOD”) when only two of the monitored transitions were observed – but again in at least two biological replicates. The only exception is the MetOx form of peptide E7_11-19_, where the intensity of third possible transition was so low that we excluded it from the analysis, thus only monitored 2 transitions, and still designated the peptide “detected”. All detected HPV16-derived peptides are listed in Table1 in the main paper. Three examples of chromatogram and MS^3^ spectra matches for a peptide detected in an IP sample and the respective synthetic reference peptide are shown in Figure 3. The remaining MS^3^ spectra matches are shown in Supplementary Figure 6.


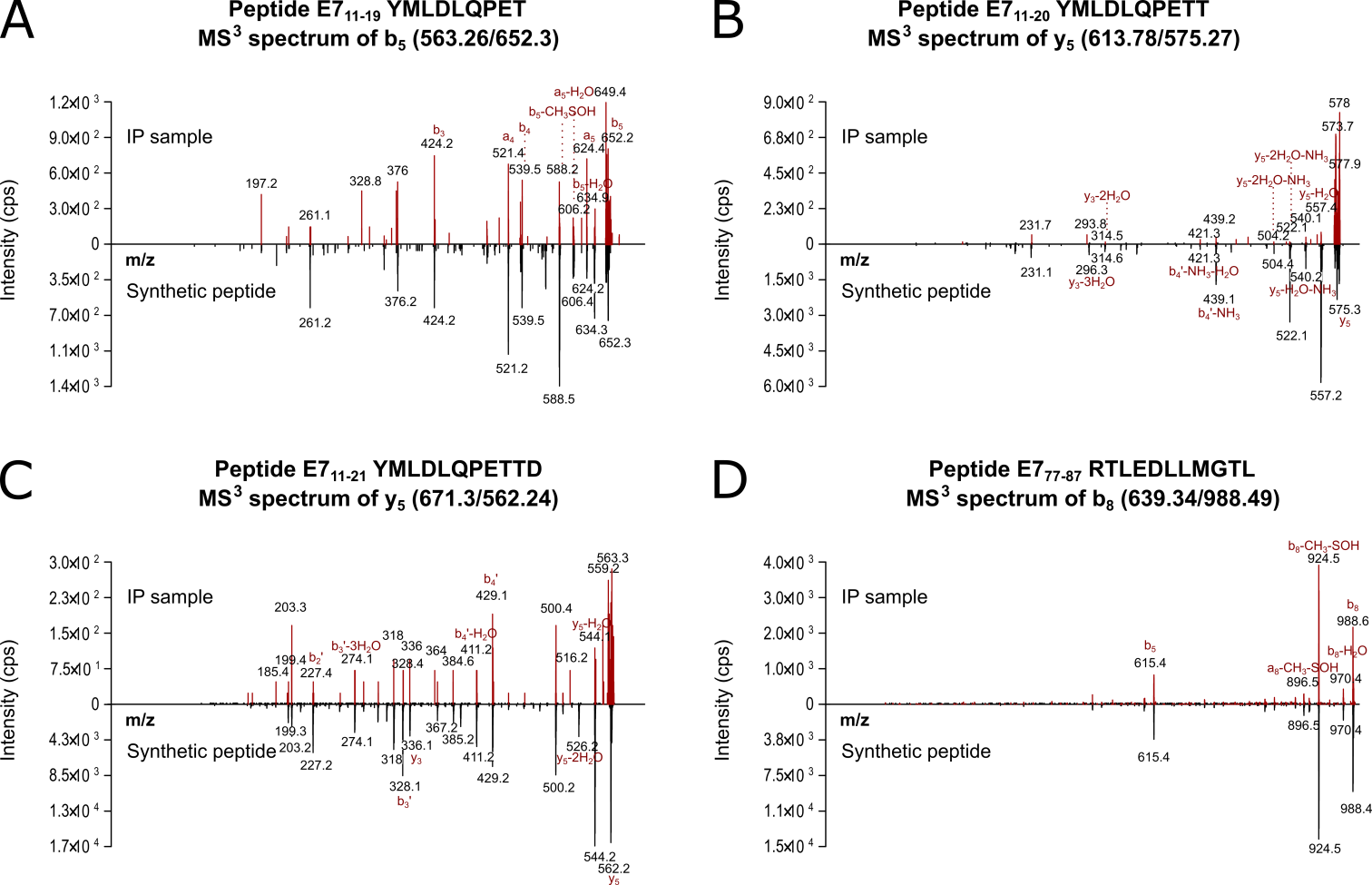


**Supplementary Figure 6: continued on next page**

**
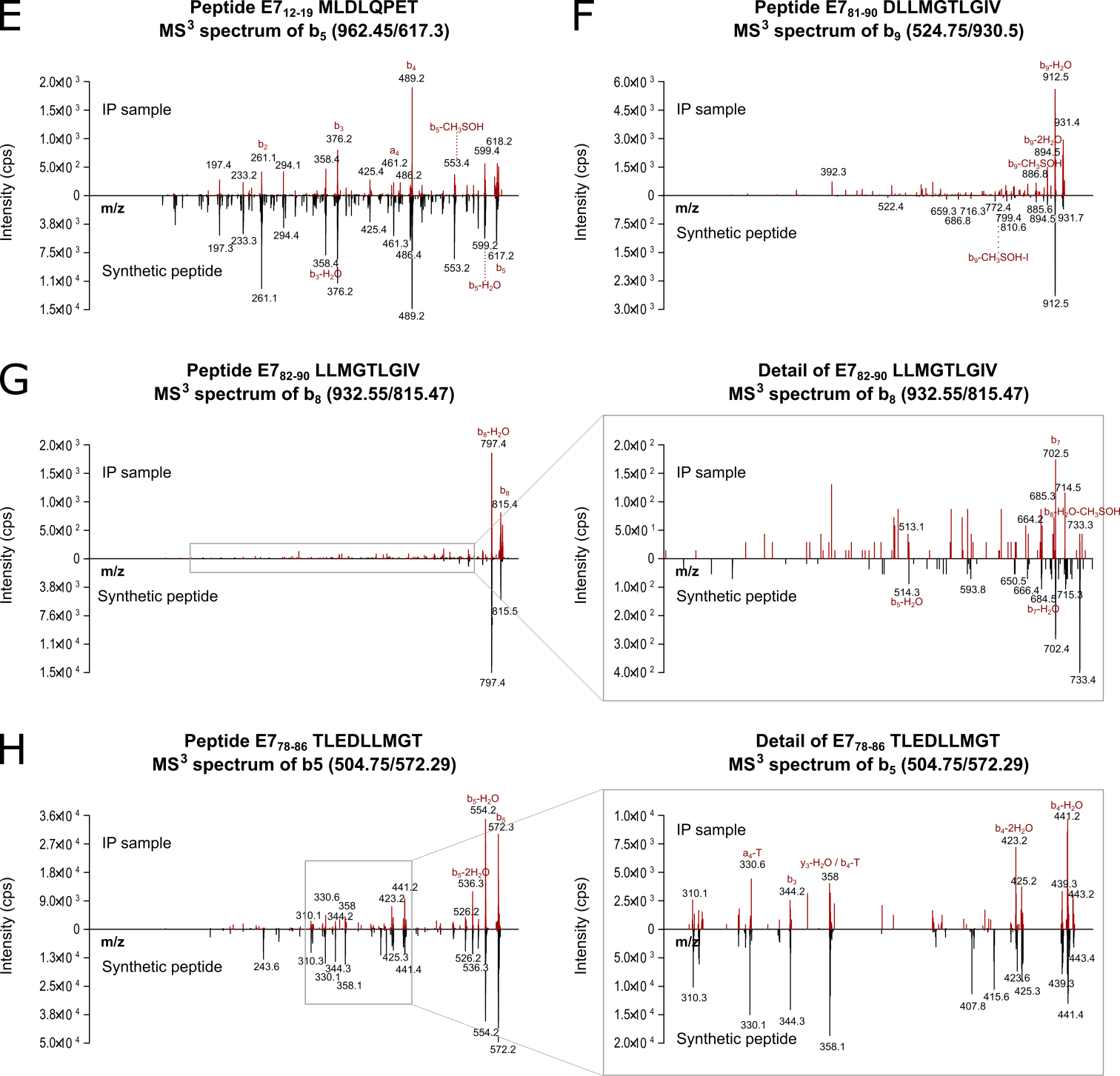
**

**Supplementary Figure 6. MS^3^ spectra for selected peptides in an IP sample and from respective synthetic peptides.** Peptide sequences and monitored transition details are indicated above each panel. CaSki IP samples were processed with Zorbax microcolumns and analyzed with LC-MS^3^. All these Met-containing peptides were detected in their oxidized form. For easier comparison, the results for the IP sample and the synthetic peptide are displayed on the same axis. Representative results of one out of at least three biological replicates are shown. m/z values are indicated in black, fragment annotations in red. T: threonine; b_x_’: MS^3^ fragments derived from a y-ion.

**References**

[1] J. Rappsilber, M. Mann, Y. Ishihama, *Nat Protoc* **2007**, 2, 1896.

[2] L. G. Falkenby, G. Such-Sanmartin, M. R. Larsen, O. Vorm, N. Bache, O. N. Jensen, *J Proteome Res* **2014**, 13, 6169.

[3] B. MacLean, D. M. Tomazela, N. Shulman, M. Chambers, G. L. Finney, B. Frewen, R. Kern, D. L. Tabb, D. C. Liebler, M. J. MacCoss, *Bioinformatics* **2010**, 26, 966.

[4] B. Schilling, M. J. Rardin, B. X. MacLean, A. M. Zawadzka, B. E. Frewen, M. P. Cusack, D. J. Sorensen, M. S. Bereman, E. Jing, C. C. Wu, E. Verdin, C. R. Kahn, M. J. Maccoss, B. W. Gibson, *Mol Cell Proteomics* **2012**, 11, 202.

[5] J. Cox, M. Mann, *Nat Biotechnol* **2008**, 26, 1367.

[6] I. V. Shilov, S. L. Seymour, A. A. Patel, A. Loboda, W. H. Tang, S. P. Keating, C. L. Hunter, L. M. Nuwaysir, D. A. Schaeffer, *Mol Cell Proteomics* **2007**, 6, 1638.

[7] W. M. Nadler, D. Waidelich, A. Kerner, S. Hanke, R. Berg, A. Trumpp, C. Rosli, *J Proteome Res* **2017**, 16, 1207.

[8] N. P. Croft, S. A. Smith, Y. C. Wong, C. T. Tan, N. L. Dudek, I. E. Flesch, L. C. Lin, D. C. Tscharke, A. W. Purcell, *PLoS Pathog* **2013**, 9, e1003129.

[9] R. Vita, J. A. Overton, J. A. Greenbaum, J. Ponomarenko, J. D. Clark, J. R. Cantrell, D. K. Wheeler, J. L. Gabbard, D. Hix, A. Sette, B. Peters, *Nucleic Acids Res* **2015**, 43, D405.
